# Pulmonary manifestations of gastroesophageal reflux disease

**DOI:** 10.4103/1817-1737.53347

**Published:** 2009

**Authors:** Gajanan S. Gaude

**Affiliations:** *Prof. and Head, Department of Pulmonary Medicine, J. N. Medical College, and Consultant Chest Physician, Prabhakar Kore Hospital & Medical Research Centre, Belgaum -590010, India*

**Keywords:** Gastroesophageal reflux disease, lungs, pulmonary

## Abstract

Gastroesophageal reflux disease (GERD) may cause, trigger or exacerbate many pulmonary diseases. The physiological link between GERD and pulmonary disease has been extensively studied in chronic cough and asthma. A primary care physician often encounters patients with extra esophageal manifestations of GERD in the absence of heartburn. Patients may present with symptoms involving the pulmonary system; noncardiac chest pain; and ear, nose and throat disorders. Local irritation in the esophagus can cause symptoms that vary from indigestion, like chest discomfort and abdominal pain, to coughing and wheezing. If the gastric acid reaches the back of the throat, it may cause a bitter taste in the mouth and/or aspiration of the gastric acid into the lungs. The acid can cause throat irritation, postnasal drip and hoarseness, as well as recurrent cough, chest congestion and lung inflammation leading to asthma and/or bronchitis/ pneumonia. This clinical review examines the potential pathophysiological mechanisms of pulmonary manifestations of GERD. It also reviews relevant clinical information concerning GERD-related chronic cough and asthma. Finally, a potential management strategy for GERD in pulmonary patients is discussed.

Gastroesophageal reflux disease (GERD) is a condition in which the esophagus becomes irritated or inflamed because of acid backing up from the stomach. The inner lining of the stomach resists corrosion by this acid. The cells that line the stomach secrete large amounts of protective mucus. The lining of the esophagus does not share these resistant features, and gastric acid can damage it. Normally, the lower esophageal sphincter prevents reflux of acid. With GERD, however, the sphincter relaxes between swallows, allowing stomach contents and corrosive acid to regurgitate up and damage the mucosa of the esophagus. GERD affects nearly one third of the adult population to some degree, at least once a month. Almost 10% of adults experience GERD weekly or daily. Not just adults, even infants and children can have GERD. [Figure 1] demonstrates the gastric acid reflux into the esophagus and trachea.

**Figure 1:**
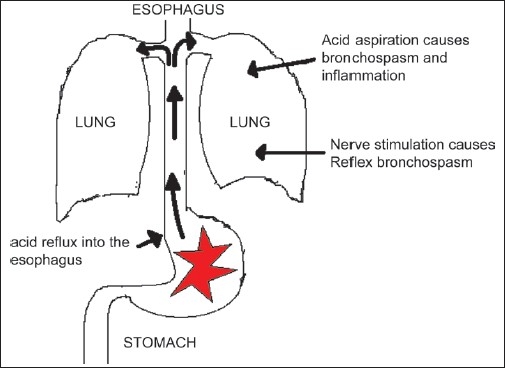
The gastric acid reflux into the esophagus and trachea

## Causes of GERD

No one knows the exact cause of gastro esophageal reflux. The following are several contributing factors that weaken or relax the lower esophageal sphincter, making reflux worse:

Lifestyle — Use of alcohol or cigarettes, obesity, poor posture (slouching).

Medications — calcium channel blockers, theophyllines, nitrates, antihistamines.

Diet — Fatty and fried foods, chocolate, garlic and onions, drinks with caffeine, acid foods such as citrus fruits and tomatoes, spicy foods, mint flavorings.

Eating habits — Eating large meals, eating soon before bedtime.

Other medical conditions — Hiatus hernia, pregnancy, diabetes, rapid weight gain.

## Pulmonary Manifestations of GERD

Historically, pulmonary manifestations have been recognized as a potential consequence of GERD. A major advance in the understanding of extra-esophageal manifestations comes from the recognition that a significant number of patients with asthma or chronic cough, particularly if it is nocturnal, have gastroesophageal reflux as a trigger.[1] Extra-esophageal symptoms of GERD are highly prevalent among patients with both frequent and infrequent typical GERD symptoms.

GERD can cause various pulmonary manifestations: Chronic cough, bronchial asthma, bronchitis, pneumonia and interstitial fibrosis [Table 1]. Out of these, chronic cough and bronchial asthma are more common manifestations of GERD, and these will be discussed in greater details in this review.

**Table 1:** Respiratory disorders associated with gastroesophageal reflux disease*

Bronchial asthma (Reflux asthma syndrome)
Chronic persistent cough (Reflux cough syndrome)
Chronic bronchitis
Pulmonary aspiration complications
(Lung abscess, bronchiectasis, aspiration pneumonitis)
Idiopathic pulmonary fibrosis
Chronic obstructive pulmonary disease
Obstructive sleep apnea syndrome

* The causal relationship between GERD and respiratory disorders is not established with the same degree of likelihood for the different manifestations

## Prevalence

According to the studies in the literature, pathological GERD can be found in 30% to 80% of patients with asthma. On the other hand, patients with esophagitis are more likely to have asthma than patients without esophagitis. In the ProGERD study,[2] the occurrence of asthma depended on longer GERD duration and was more prominent in male and older subjects. The kind of GERD disease, weight and gender did not have significant relationship with asthma.[1] A recent systematic review[3] of 28 epidemiological studies found a 59.2% weighted average prevalence of GERD symptoms in asthmatic patients, compared to 38.1% in controls. The corresponding prevalence of asthma in GERD patients was 4.6%, compared to 3.9% in controls. One longitudinal study showed a significant association between a diagnosis of asthma and a subsequent diagnosis of GERD, whereas the two studies that assessed whether GERD precedes asthma gave inconsistent results.[3] The prevalence of reflux symptoms was similar (75%) in a subgroup of patients with difficult-to-control asthma.[4] A large population-based epidemiologic investigation showed that young adults with nocturnal reflux symptoms had a higher prevalence of asthma and respiratory symptoms as compared with patients without reflux symptoms.[5] Another study by Sontag *et al.*[6] showed that asthmatics had more frequent and more severe daytime as well as nighttime reflux symptoms and suffered from more reflux-related nocturnal awakening from sleep. Based on continuous ambulatory esophageal pH-monitoring, at least 50% of adults and children have evidence of GERD.

The prevalence of GERD-associated cough ranges from 10% to 40%, depending on the patient population, type of diagnostic test used and whether more than one etiology of cough is ascertained. An epidemiological association between GERD and chronic cough has been reported in patients of all age groups[7]. Patients with nocturnal reflux may be at higher risk of respiratory symptoms in general, and of cough in particular.[8] However, cough can simultaneously be on account of more than one condition, and it is frequently associated with other respiratory disorders, especially asthma or laryngopharyngeal manifestations such as laryngitis.[9] The most convincing evidence linking reflux and cough comes from pH or pH-impedance–monitoring studies. Harding *et al.,*[10] using pH-monitoring, observed a strong correlation between esophageal acid events and respiratory symptoms in asthmatics with GERD symptoms and abnormal acid exposure, with almost all cough episodes associated with pH value of less than 4. It was observed that even in patients without reflux symptoms but with abnormal pH-monitoring values, 72% of cough events were associated with esophageal acid events. In another study,[11] GERD was found to be the cause of chronic cough in up to 10% of patients when the diagnosis was made by history, endoscopy or barium esophagogram. Adding 24-hour esophageal pH testing in the diagnostic armamentarium, GERD can account for chronic cough in up to 40% of patients. In children, the prevalence of GERD as a cause of chronic cough is reported to be 4% to 15%.[12] With the use of stringent criteria, Blondeau *et al.*[13] found that acidic reflux was a potential mechanism of cough in 23% of patients; and weak acidic reflux contributed to cough in another 17% of the patients.

## Pathogenesis

GERD can be a compounding factor in the control of asthma. Not only is the asthmatic patient more likely to have GERD as compared to the general population, but also GERD is recognized as a potential trigger in many cases of severe asthma.[14] Bronchial asthma may itself favor the development of reflux by several mechanisms. Pulmonary hyperinflation contributes to the diaphragmatic dysfunction; and bronchoconstriction results in an increase in negative pleural pressure, which effects a change in the pressure gradient between the thorax and the abdomen. Furthermore, frequent use of bronchodilators may contribute to a decrease in lower esophageal sphincter tone.[15] Several mechanisms have been implicated by which GERD may exacerbate the preexisting asthma. Two mechanisms are important in understanding this exacerbation of asthma[16]: [i] esophageal acid stimulates vagally mediated tracheobronchial responses, and this increases the bronchial hyper-responsiveness to other stimuli; [ii] by irritating sensitive asthmatic airways following micro-aspiration of even tiny refluxed material into the tracheobronchial tree, which contributes to the adverse airway effects. Central nervous system reflex pathways as well as local axon reflexes may each contribute to the pathogenesis of both asthma and GERD. When activated, airway nociceptors precipitate defensive reflexes such as cough, bronchospasm and mucus secretion. Nociceptors innervating both the airways and the esophagus respond to similar stimuli with defensive maneuvers. The synergistic interactions between esophageal nociceptors and airway sensory nerves may precipitate asthma-like symptoms associated with GERD.[14] It has also been observed that abrupt decrease in tracheal pH coincides with broncho-constriction during episodes of gastroesophageal reflux in patients with asthma and typical GERD symptoms.[9]

**Figure 2:**
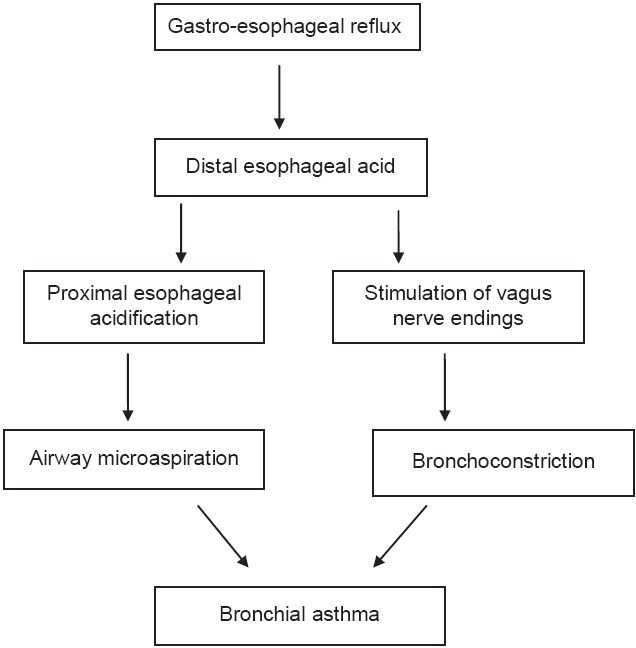
Mechanism of a linkage between asthma and GERD

Figure 2 summarizes the current concepts of theories that explain a link between asthma and GERD. The reflux theory suggests that symptoms of asthma are due to reflux of acid into the esophagus followed by aspiration into the proximal airways. Animal studies have proven that once trachea is acidified, there is a demonstrable increase in airway resistance. This is confirmed by scintigraphic demonstration of aspiration of radio-labeled isotope into the airway in some patients with GERD and respiratory symptoms.[3] Another theory suggests that distal esophageal acidification results in vagal stimulation and consequent broncho-constriction, independent of airway micro-aspiration.[9] This theory gains support from the observation that not all patients who develop bronchospasm have demonstrable proximal esophageal acidification. Further, even among those who show abnormal proximal esophageal pH, there is improvement in respiratory symptoms with control of distal gastroesophageal reflux alone. It is also possible that physiological changes in asthma, including increased lower esophageal pressure, the mechanical influence of a depressed diaphragm caused by hyperinflation, and cough mediated by increased abdominal pressure, may contribute to gastroesophageal reflux to some degree. In addition, some of the medications used for treatment can aggravate gastroesophageal reflux; thus, there is a perception that gastroesophageal reflux may be an effect rather than cause of chronic respiratory conditions.

There are 2 different mechanisms by which GERD can cause chronic cough: (i) acid in the distal esophagus stimulating a vagally mediated esophageal tracheobronchial cough reflex and (ii) micro- or macro-aspiration of esophageal contents into the larynx and tracheobronchial tree. One study proposed that gastro esophageal reflux causes esophago-tracheo-bronchial reflex. Using dual-probe 24-hour esophageal pH-monitoring, it has been observed that reflux occurred simultaneously in 78% and within 5 minutes in 90% of cough episodes.[16] It is proposed that the reflex arc is the likely mechanism by which GERD leads to cough in these patients; although intra-esophageal pH may not be the sole mediator. Micro-aspiration of the contents from the proximal esophageal refluxate can also result in GERD-related cough and can account for up to 10% to 15% of cases of unexplained chronic cough. It results from smaller-volume refluxate and produces laryngeal inflammation with or without bronchial inflammation. The resulting inflammation due to mucosa damage leads to cough and hoarseness in such patients, without producing the classical symptoms of GERD. There is also evidence that cough might produce GERD, probably by increasing the pressure gradient between the thorax and abdomen or by causing transient lower esophageal sphincter relaxation.[17]

## Clinical Manifestations

Many patients with asthma report GERD symptoms, including heartburn, regurgitation and dysphagia. Some patients may have clinically silent GERD, especially in the context of difficult-to-treat asthma.[18] A high degree of esophageal dysfunction has also been reported among patients with asthma, including esophageal dysmotility, lower esophageal sphincter hypotension and a positive Bernstein test. Specific esophageal motility abnormalities in asthma patients include ineffective esophageal motility, with a reported prevalence of 53.3%; nutcracker esophagus in 7.6%; and low lower esophageal sphincter pressure in 15.4% of the patients.[19] Endoscopy might also reveal esophagitis or Barrett's esophagus among patients with asthma, although most will have esophagitis[20] Compared with normal controls, patients with asthma have a higher frequency of reflux symptoms; most frequent lower esophageal sphincter hypotension, by manometry; and increased esophageal acid contact duration, by 24-hour pH-monitoring, all of which further support the association between GERD and asthma.[9]

The most common causes of chronic cough are postnasal drip, asthma and GERD. One should be able to predict cough due to GERD in the following categories of patients: Those not taking ACE inhibitor, nonsmokers, those with normal chest x-ray, those with negative broncho-provocative test for asthma, and those with persistent cough despite effective treatment for postnasal drip.[21] A study confirmed that patients with cough and GERD have significantly reduced laryngopharyngeal sensitivity to air stimuli compared with healthy subjects, which could potentially result in an increased risk of aspiration. Exposure to small amounts of acid was found to significantly impair the sensory integrity of the laryngopharynx.[22]

## Diagnosis of Pulmonary Manifestations of GERD

The patient's history is an extremely important part of the diagnosis of GERD-associated asthma. The diagnosis is important to consider, however, because significant improvement in symptoms and in asthma control occurs with appropriately treated GERD.[16] Certain clinical clues can be helpful in identifying GERD-related asthma. Patients' symptoms suggesting reflux include nocturnal cough, worsening of asthma symptoms after eating large meal, drinking alcohol, or being in the supine position. GERD should be considered in asthmatics who initially present in adulthood, in those without an intrinsic component and in those not responding to bronchodilator or steroid therapy. An additional clue may be the development of reflux symptoms before the onset of asthma, or heartburn heralding an asthma attack. Patients with chronic cough should have a history taken and physical examination carried out to evaluate common causes of cough (asthma, sinusitis, GERD, ACE inhibitors), as well as chest radiograph. GERD should be considered if there are typical gastrointestinal symptoms or if cough remains unexplained after standard investigations. The diagnosis of GERD as the cause of cough can only be made with certainty when cough subsides with specific anti-reflux therapy.

Esophageal tests that may be helpful in diagnosis include the barium esophagogram, gastroesophageal scintigraphy, and prolonged esophageal pH–monitoring. Esophageal pH- monitoring is considered the gold standard for the diagnosis of GERD and is the only esophageal test that can directly correlate acid reflux episodes with wheezing or other symptoms of bronchospasm. Esophageal pH–monitoring in patients with suspected GERD-related manifestations may represent a more accurate approach.[23] However, the diagnostic yield of esophageal pH–monitoring in patients with respiratory symptoms is probably far from perfect. Sensitivity is improved by using a combination of pH- and impedance-monitoring, but this technology has not yet been standardized to a level which would satisfy the definition of a test suitable for routine clinical practice. The problem is further complicated by the fact that GERD is a very common disorder which may coexist with respiratory symptoms only by chance, i.e., without any causal relationship. A much more relevant approach is to demonstrate that there is a reliably positive association between the onset of a given respiratory symptom and the occurrence of a reflux episode. Gastroesophageal scintigraphy has a high specificity but low sensitivity, which limits its usefulness. Moreover, approximately two thirds of the patients affected with asthma responded favorably to anti-reflux therapy.[9]

## Management of GERD in Patients with Pulmonary Manifestations

Treatment for GERD is aimed at reducing the abnormal backflow, or reflux of acid, into the esophagus; preventing injury to the esophagus or helping it to heal if injury has already occurred; preventing GERD from recurring; and preventing complications of GERD.

### Initial treatment

Treatment for people who have symptoms of GERD begins with making lifestyle modifications. Sleeping with an elevated head in patients with documented nighttime reflux episodes, smoking cessation, weight reduction and low-fat diet (< 45g/d) have all been found to be useful measures. Avoidance of food and beverages with a pH of < 5 and/or capability of relaxing the lower esophageal sphincter — such as alcohol, chocolate, mint, onions, tea, cola, citrus fruits — is also highly recommended; and patients should avoid food and beverages 2 to 3 hours before going to the bed.[24] Medications for GERD include proton pump inhibitors (PPIs) and H2 blockers. Patients may require different medications or combinations of medications before finding the one that best relieves their symptoms. Long-term medication therapy is usually necessary to treat severe, persistent symptoms or complications of GERD. If GERD symptoms are present, begin a 3-month empiric twice-daily trial of PPI, while monitoring baseline respiratory symptoms, pulmonary symptoms and peak expiratory flow rate. If at the end of empiric trial, pulmonary symptoms do not improve, then 24-hour esophageal pH–testing should be performed while the patient is on GERD regimen, to see if acid is adequately suppressed. If the patient's respiratory symptoms are found to improve after a 3-month empiric trial, then maintenance GERD therapy is required.

### Role of proton pump inhibitors in the management of GERD-related asthma and chronic cough

Proton pump inhibitors are the most effective acid-suppression medications available and are the cornerstone of therapy for GERD and other acid-mediated conditions. Once-a-day PPI therapy improves chronic cough in patients with GERD, and the effect of PPI in ameliorating both cough and reflux symptoms continues after treatment ceases. There is evidence that 2 months with PPI is sufficient to reduce cough in patients with GERD.[2] Jaspersen[1] found that GERD-related cough nearly always improves with PPI treatment. Available evidence supports the use of PPI with doubled standard doses for at least 12 weeks in GERD-related cough.[25] A trial of PPI therapy is increasingly being considered a first-line diagnostic test in those with suspected reflux-related extra-esophageal symptoms.[26] A recent Cochrane systematic review[27] retrieved 13 randomized, controlled trials of GERD treatment for cough without lung disease in children (3 trials) and adults (10 trials). Six studies comparing PPI treatment (2 or 3 months) with placebo were included and analyzed on an intention-to-treat basis. Overall, there was no significant difference between placebo and PPI (odds ratio = 0.46; 95% CI, 0.19–1.15) in the resolution of cough. These results are consistent with those of a large randomized controlled trial conducted in patients with laryngeal reflux and ENT manifestations which failed to show any benefit of esomeprazole 40 mg for 16 weeks compared to placebo.[28] However, regarding the specific cough meta-analysis, further sensitivity analysis showed significant changes in cough scores in those receiving PPI in cross-over trials. Two trials showed improvement in cough after only 5 days to 2 weeks of treatment. Since there are insufficient data to draw any conclusions by meta-analysis or from large, randomized, controlled trials that have included poorly selected patients, it should not be concluded too quickly that GERD treatment is not effective in subgroup of patients with reflux-related cough. Chang *et al.*[29] concluded in their meta-analysis (including only 11 trials) that use of a PPI to treat cough associated with GERD has some effect in some adults. Impedance-pH monitoring with careful analysis of the symptom-reflux temporal relationship may help to select the right patients who can truly benefit from treatment of GERD.[30,31] Based on the previous data, 2 different strategies for management of patients with suspected reflux-related cough have been proposed.[32] The empirical strategy with PPI (usually double dose) given for at least 3 months is probably the most popular one, but it should be underlined that this strategy is not supported by strong evidence. The second strategy consists of investigations, which should ideally detect both acidic and non-acidic reflux. Patients who failed to respond to empirical therapy should be investigated. In the event of negative results, it is preferred to avoid PPI therapy and eventually to repeat pH-impedance monitoring after 6 to 12 months of follow-up.[32] A recent long-term clinical study has shown that the majority of patients with chronic cough who were evaluated had improved after 2 years of therapy.[33]

With regard to medical therapy of GERD and asthma, studies using PPIs have had more encouraging results than those using antacids or H2 receptor antagonist. The latter have yielded inconsistent results on asthma symptoms and peak expiratory flow rates (PEFRs). Numerous clinical trials[34–41] have investigated the effects of anti-reflux therapy on asthma control [Table 2]. A systematic review of all published trials concluded that medical treatment for GERD improved asthma symptoms in 69% of the patients, reduced asthma medication use in 62%, and improved PEFRs in 26% of the patients.[42] Gibson *et al.*[43] performed a systematic review of 12 randomized, placebo-controlled trials using the Cochrane methodology, and concluded that there was no overall improvement in asthma following treatment for GERD; and it pointed out, as others have done,[44] many flaws in study designs and methodologies, so no definite conclusions could be drawn. More recent studies have aimed at focusing on patients with GERD and/or difficult-to-treat asthma. One study[35] using PPI to treat patients with asthma and GERD over a period of 3 months showed that 73% of patients experienced marked alleviation of asthma symptoms or increases in PEFRs. Treatment reduced asthma symptoms by 57% after 3 months. The patients most likely to benefit from the therapy were those with frequent regurgitation or excessive proximal esophageal acid reflux.

**Table 2:** Treatment of asthma — Randomized controlled studies with proton pump inhibitors published during the last 10 years

Author	Number of patients	Treatment	Asthma symptoms	PEFR	FEV_1_
Shimuzu *et al*[37]	30	Lanzoprazole 30 mg for 8 weeks	Improved	Improved	Unchanged
Kiljender *et al*[40]	107	Omeprazole 40 mg for 8 weeks	Improved*	Unchanged	Improved
Kiljender *et al*[35]	770	Esomeprazole 80 mg for 16 weeks	NA	Unchanged@	NA
Stordal *et al*[38]	38	Omeprazole 20 mg for 12 weeks	Unchanged	NA	Unchanged
Boeree *et al*[37]	36	Omeprazole 80 mg for 12 weeks	Unchanged	Unchanged	Unchanged
Littener *et al*[39]	207	Lansoprazole 60 mg for 24 weeks	Unchanged$	Unchanged	Unchanged
Jiang *et al*[41]	30	Omeprazole 20 mg and domperidone 30 mg for 6 weeks	Improved	Improved	Improved

NA: Not available;

* Nighttime asthma symptoms only;

@ Improvement in subgroup of patients with nocturnal respiratory symptoms and GERD;

$ significant reduction of asthma exacerbations and improved quality of life

In a cohort of difficult-to-control asthmatics, PPI and prokinetic therapy for 7 weeks resulted in complete improvement of symptoms in 86%, moderate improvement in 11% and lack of improvement in only 4% of the patients.[35] Symptoms reappeared rapidly following discontinuation of GERD therapy. Meta-analysis of 12 studies over a 30-year period assessed the effect of medical anti-reflux therapy on asthma control and found that almost 70% of over 300 subjects had an improvement in asthma symptoms.[42] Other studies have documented improvement in objective parameters of pulmonary function with treatment of GERD.[45] There is also improvement in quality of life and symptom scores with abolition of reflux.[10] It has also been observed that control of GERD improves morbidity and reduces need for asthma therapy.[46] PPI treatment also improves nocturnal asthma symptoms.[47] There is evidence that more severe GERD might predict a more favorable asthma outcome with PPI therapy.[48] For effective management of GERD-related symptoms, PPIs should be used at a dose double that of the standard dose for a minimum of 2 to 3 months.[49] All the PPIs have shown varying degree of results in GERD patients with asthma.[50] Rabeprazole achieves more potent acid suppression than other PPIs.[51] In a clinical practice setting, rabeprazole provided rapid relief of symptoms, confirming results seen in controlled trials.[52] In summary, the interactions between GERD and asthma are complex; however, the anti-reflux treatment is appropriate in some asthmatics, but the target population should be more precisely defined in the future. In clinical practice, the patients presenting with difficult-to-treat asthma and/or nocturnal symptoms without GERD symptoms should be offered an esophageal pH study off thera py to detect “silent GERD.” Patients presenting with typical GERD symptoms and/or abnormal pH study results should be treated with a 3-month double-dose PPI therapy.

### Other respiratory problems

Unlike asthma and cough, in which the esophago-bronchial reflex may play an important role, direct aspiration of gastric contents into the lung is thought to be the major patho-physiological mechanism in other respiratory disorders. [53] Epidemiological studies in patients with reflux esophagitis have shown a slightly increased risk for idiopathic pulmonary fibrosis (IPF; OR = 1.36), chronic bronchitis (OR = 1.28), chronic obstructive pulmonary disease (OR = 1.22) and pneumonia (OR = 1.15).[54] Recently, it has been demonstrated that chronic obstructive pulmonary disease (COPD) exacerbations in patients with GERD symptoms are twice as high as in those without GERD symptoms.[19] Micro-aspiration of gastric contents and/or vagal irritation from gastro esophageal reflux may constitute airway irritants and thus represent a potential pathogenic mechanism for acute exacerbations of COPD. The impact of GERD on exacerbations of COPD had never been evaluated earlier. COPD exacerbations were twice as high in patients with GERD symptoms as in those without GERD symptoms (3.2 per year vs. 1.6 per year; P =.02). The study concluded that the presence of gastroesophageal reflux symptoms is associated with increased exacerbations of COPD, and these patients are twice as likely to be hospitalized, have an emergency department visit or unscheduled clinic visit when compared with COPD patients with less frequent GER symptoms.[19] They also observed that the use of anti-reflux therapy did not have a protective effect against exacerbations, highlighting the importance of multi-drug therapy in patients with COPD exacerbations. In patients with IPF, high prevalence of reflux has been observed compared to the general population.1 [55] However, there are no published trials showing that anti-reflux therapy could influence any parameter of pulmonary function or respiratory symptoms in IPF. Recently, attention has also been drawn to the relationship between obstructive sleep apnea syndrome (OSAS) and GERD.[56–61] OSAS is a condition characterized by pharyngeal narrowing and upper airway obstruction during sleep that results in repeated episodes of decreased oxygen saturation and brief arousals. OSAS and GERD are very common disorders, which share the same risk factors (such as increased body mass index) and thus may coexist in the same subject. However, an increased number of reflux episodes and excessive esophageal acid exposure were reported in patients with OSAS, as compared to patients without the syndrome.[57] The relationship between OSAS and GERD remains uncertain, and further mechanistic studies are required to elucidate the link between these two phenomena. It has been suggested that OSAS may predispose patients to nocturnal reflux, because apneic episodes are associated with increased arousals, transdiaphragmatic pressure changes and low intra-thoracic pressures. Recently, obesity has been suggested as a common pathophysiological mechanism of GERD and OSAS.[61] Finally, the causality of the relationship between OSAS and GERD was further supported by the results of a therapeutic trial[58] showing benefit of treatment with continuous positive airway pressure on GERD symptoms. On the other hand, PPI therapy may improve OSAS outcome, as shown by Bortolotti *et al.*,[60] but such a small-sample size trials warrants further confirmation. Severe pulmonary complications of GERD, usually resulting from pulmonary aspiration, often occur in children with neurological diseases and/or debilitating underlying medical conditions.[62] However, even in children without neurological defects, there is a significant association between GERD and pneumonia and bronchiectasis.[63] Recently, the use of pH-impedance monitoring has allowed investigators to revisit the evaluation of the relationship between acidic reflux, non-acidic reflux and apnea in infants. One study[64] showed the limitations of pH-monitoring in detecting the temporal association between reflux and apneic episodes because of the buffering effect of feeding in infants; in contrast, the majority of reflux-related apneas were associated with non-acidic reflux, more accurately detected by the impedance technique. However, a study by another group failed to detect any significant relationship between reflux and apnea of prematurity.[65] Recently, it has been reported that in patients with end-stage lung disease awaiting lung transplantation, symptoms are insensitive and nonspecific for GERD diagnosis.[32] In contrast, esophageal motility is frequently abnormal and acidic reflux is common, reaching the proximal esophagus in approximately 50% of these patients.[66] The role of reflux may be important (among other factors) in the development of the bronchiolitis obliterans syndrome, thus favoring graft dysfunction.[67]

### Role of Surgery in GERD

Fundoplication surgery is the most common surgery used to treat GERD. It may be used to treat GERD symptoms that have not been well controlled by medications. In fundoplication surgery, the fundus of the stomach is wrapped around the esophagus and sewn into place to strengthen the lower esophageal sphincter. Some relatively new nonsurgical procedures used to treat GERD are still undergoing trials to determine their long-term safety and effectiveness. These include Stretta radiofrequency procedure, in which radiofrequency energy is delivered through an endoscope to tighten the lower esophageal sphincter; and EndoCinch procedure, in which an endoscopic sewing device is used to make a series of sutures that adjusts the lower esophageal sphincter so that it blocks acid reflux more effectively.

**Figure 3:**
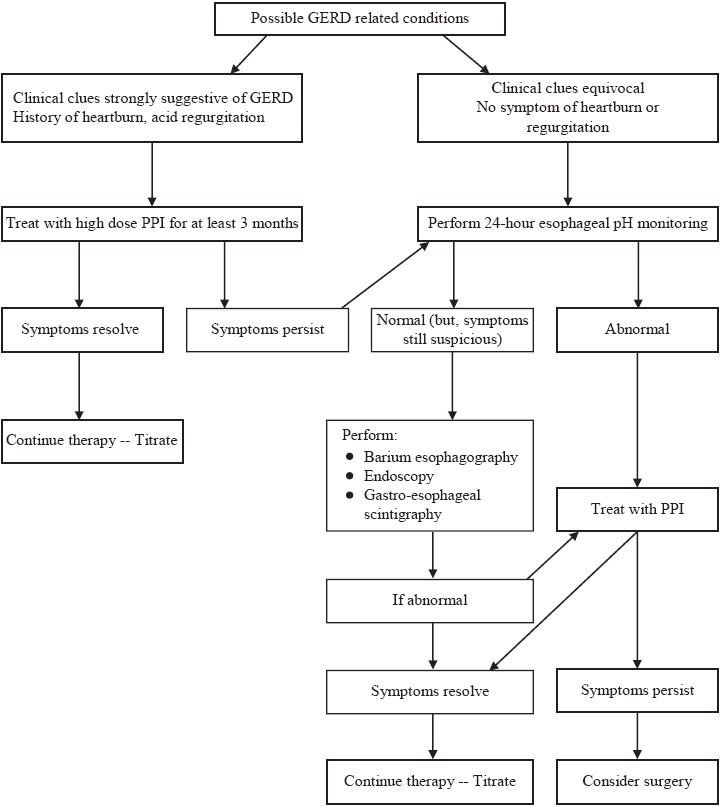
Approach to diagnosing and managing GERD-related extra-esophageal symptoms

Many uncontrolled studies have been carried out to investigate the effect of anti-reflux surgery on asthma outcome. The results of these studies suggest that surgery could improve asthma symptoms and reduce medication use in 80% to 90% of the asthmatics; and pulmonary function, in approximately 25%.[68] The only reported controlled studies have compared H2-receptor antagonists and fundoplication. In the first study, which was with cimetidine, drug therapy and surgery were both associated with an improvement in asthma symptoms and medication requirement at 6 months, but not with improved pulmonary function, as compared with placebo treatment.[69] The second study, which was with ranitidine, showed that 74.9% of patients in the surgery group improved as compared with 9.1% of the medical group and 4.2% of the control group.[70] However, although no data are yet available regarding the prevalence of biliary and/or non-acidic reflux in asthma, surgery may be considered, theoretically, to be a more effective method of avoiding any type of GERD.[32] In patients with chronic cough and documented reflux who fail to respond to acid suppression, anti-reflux surgery may be indicated for long-term control in well-selected patients.[71] This may also be the case for patients with refractory acidic or non-acidic reflux and a well-documented correlation between reflux episodes and cough. Figure 3 summarizes the management of GERD and its pulmonary manifestations.
